# Enhancement of Tribological and Mechanical Properties via Soda-Lime Silica Glass Reinforced Al-Mg-Zn Matrix Composite

**DOI:** 10.12688/f1000research.183177.2

**Published:** 2026-08-20

**Authors:** Babuli Chandra Kar, Sambit Kumar Mohapatra, Pushkar Jha

**Affiliations:** 1 School of Mechanical Engineering, Kalinga Institute of Industrial Technology (KIIT) Deemed to be University, Bhubaneswar, Odisha, 751024, India

**Keywords:** Al-Mg-Zn alloy, Soda-Lime Silica Glass, Metal Matrix Composite, Wear Resistance, Tribology.

## Abstract

**Background:**

A large volume of waste Soda-Lime Silica (SLS) glass has become a major environmental concern due to improper disposal and limited recycling utilization. At the same time, the increasing industrial demand for high-strength-to-weight ratio materials has opened opportunities for incorporating SLS glass as reinforcement in aluminium matrix composites.

**Methods:**

In this study, a novel aluminium, magnesium, zinc and SLS glass hybrid metal matrix composite (MMC) was developed, using an ultrasonic vibration-supported stir-squeeze casting process. Glass reinforcements of 1, 2, 4, and 5 wt.% were incorporated to investigate their influence on the microstructural, mechanical, and tribological properties of the composite. The Scanning Electron Microscopy (SEM), Strain Rate Jump (SRJ) Gleeble compression test, micro-hardness, impact, pin-on-disc dry sliding wear test, wear morphology were conducted for comprehensive characterization of the cast specimen.

**Results:**

SEM pictures revealed the uniform dispersion and strong matrix-reinforcement bonding. The hardness of the fabricated composite increased by ~ 23% as the reinforcement content increased from 1 to 5 wt.%, whereas the toughness reduced by ~ 46%. The SRJ test conducted using a Gleeble thermo-mechanical simulator clearly illustrates lower compressive strength for the composites reinforced with 4 wt.% and 5 wt.% SLS glass, whereas the sample having 2 wt.% reinforcement exhibited the highest strain-rate sensitivity. The 2 wt.% composite exhibited the lowest wear during the pin-on-disc dry sliding wear test, which can be attributed to the synergistic effects of Zn and Mg, along with the strengthening imparted by the well-dispersed glass particles. At higher reinforcement percentages, micro-fracturing and wear damage became more pronounced.

**Conclusions:**

Overall, the composite reinforced with 2 wt.% SLS glass offered the best balance of strength, toughness, and wear resistance. By valorising waste SLS glasses, these hybrid composites can be utilized for lightweight, structural and wear-critical applications, thereby advancing sustainable manufacturing.

## 1. Introduction

The increasing demand for high strength-to-weight ratio materials in aerospace, defence, automotive, sports, and structural applications has promoted aluminium-based composite research. Though pure aluminium and its alloys are light in weight and resist corrosion, they usually do not have high-temperature performance. To address this, ceramic particulate-reinforced hybrid composites have gained attention in recent decades. ^1^ ^–^ ^3^ Among various production methods for particulate-reinforced composites, the liquid metallurgy route is mostly preferred for its simplicity, scalability, and cost-effectiveness. ^4^ ^–^ ^6^ This method ensures reasonably homogeneous reinforcement dispersion in the molten matrix, facilitates interfacial bonding, and grain refinement by controlled cooling, promoting it well-suited for developing high-performance hybrid composites. ^3^ ^,^ ^7^ ^,^ ^8^

The addition of magnesium (Mg) ^9^ and zinc (Zn) to aluminium matrices has been reported to enhance tribo-mechanical properties by enhancing the particle–matrix wettability, solid solution strengthening, and precipitation hardening. ^10^ ^,^ ^11^ Al–Mg–Zn composites reinforced with TiC have demonstrated improved tribological properties and increased strength. In a different system, glass particle additions in Al–Zn–Mg alloys show accelerated precipitation behaviour, which influences ageing kinetics. ^12^ Metallic glass reinforcements have been reported to have enhanced compressive strength in Al and Mg-based composites, owing to their high strength and metallic bonding capability with aluminium matrices. Soda-lime silica (SLS) glass, a low-cost, hard reinforcement, thermally and chemically stable, has been explored for enhancing wear resistance in Al alloy composites ^13^ ^,^ ^14^; however, its practical utility is limited by challenges in uniform particle dispersion and the tendency to create localised brittleness, inhomogeneous property distributions. ^15^ ^,^ ^16^ Many research exists on different ceramic reinforcements such as Al _2_O _3,_ ^17^ ^,^ ^18^ TiC, AlN, TiN, SiC ^19^ ^,^ ^20^ or B _4_C ^21^ ^,^ ^22^ etc. in Al–Mg–Zn matrix; however, the investigations including Zn, Mg, and SLS glass particulates remain scarce, highlighting a research gap in this hybrid composite system. ^23^ The comprehensive characterization and evaluation of tribo-mechanical properties of SLS glass reinforced composites fabricated using a vibration-assisted squeeze casting process are limited in existing research works. This constitutes a research gap which was addressed in the present work.

This work aims to produce Al-Mg-Zn-SLS glass hybrid MMC by varying 1,2,4, and 5 wt.% of SLS glass reinforcement via vibration-supported stir-squeeze casting process. The novelty of this work lies in the investigation of the influence of reinforcement content on strength, ductility, and wear resistance, supported by microstructure-property correlations.

## 2. Materials and methods

### 2.1. Raw materials

Commercially available pure aluminium (Al) ingots were procured indigenously and used as the base material of the composite. Magnesium (Mg, ≥99.5% purity) and zinc (Zn, ≥99.9% purity) powders were obtained from Loba Chemie Pvt. Ltd., Mumbai, India. The reinforcement, SLS glass powder, was prepared by mechanical milling of waste glassware to achieve a particle size below 45 µm. The chemical composition of the prepared soda-lime silica glass powder, determined by energy-dispersive X-ray spectroscopy (EDX), consisted of SiO₂ (73.1 wt.%), Na₂O (13.8 wt.%), CaO (8.7 wt.%), along with minor constituents such as MgO (1.2 wt.%), Al₂O _**3**_ (1.0 wt.%), and K₂O (0.8 wt.%). Following the compositions presented in Table 1, four different types of specimens were considered for fabrication.

**Table 1.** Composition selected for the specimen preparation.

Specimen type	Al (Wt.%)	Mg (Wt.%)	Zn (Wt.%)	Soda lime silica glass (Wt.%)
1	92	5	2	1
2	91	5	2	2
3	89	5	2	4
4	88	5	2	5

### 2.2. Composite fabrication process

**Fig 1.**
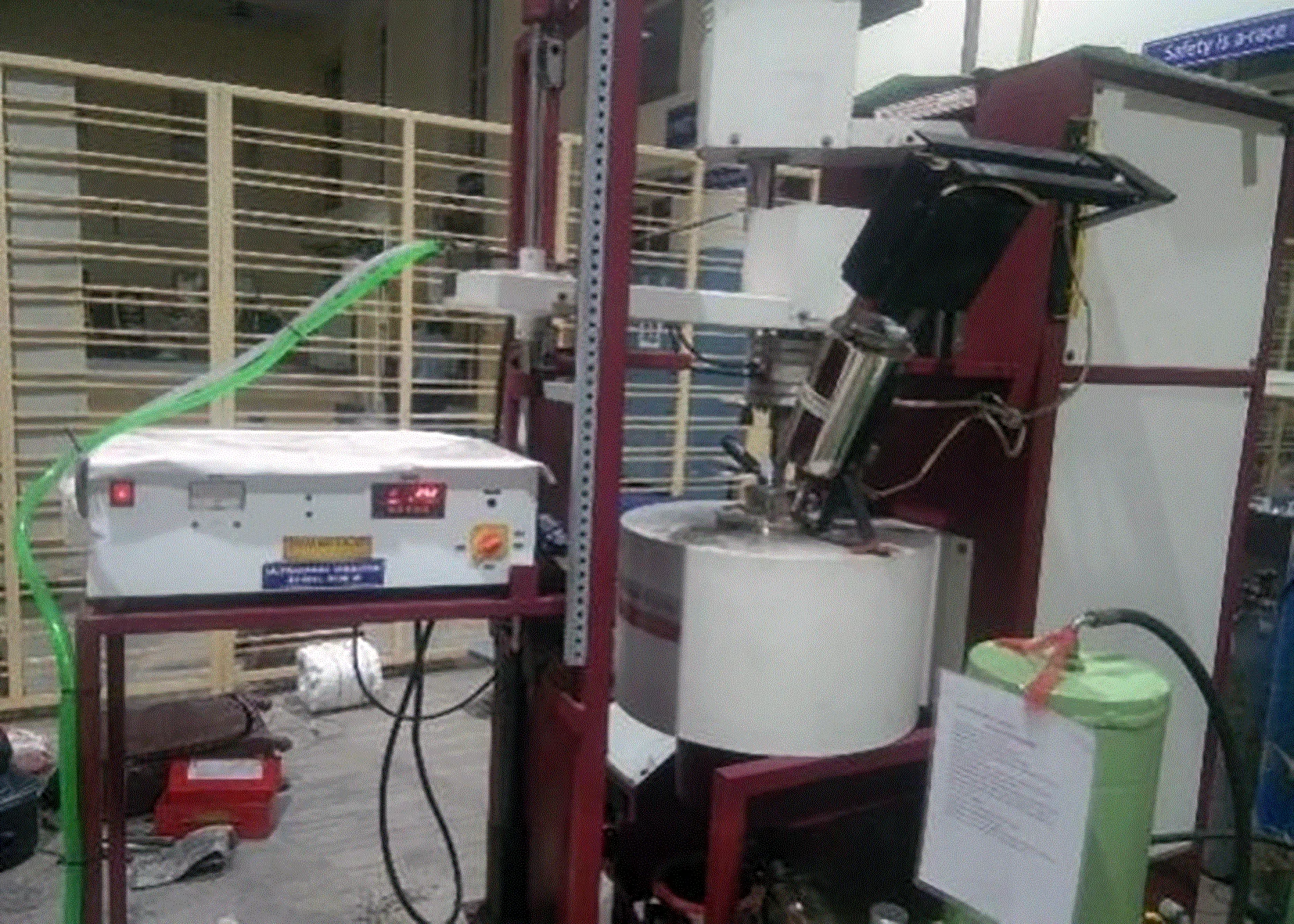
Stir-casting unit during fabrication.

The Al, Mg, Zn matrix composites reinforced with SLS glass particles were fabricated using a conventional liquid casting route. The stir casting setup was facilitated with ultrasonic vibration (20 Hz for 10 minutes) to degas as well as to improve wettability, and a squeeze setup to get a compressed defect free high dense specimen. The aluminium was melted in a graphite crucible in a resistance heating furnace at a temperature of approximately 800 ± 10 °C. Argon and SF6 gas mixture of 3 L/min was continuously purged into the melt chamber to minimize oxidation during melting and processing. Preheated Mg, Zn, and SLS glass powders at 350 °C to eliminate moisture, to avoid thermal shock and to enhance wettability were subsequently added to the molten aluminium and stirred thoroughly to ensure complete dissolution and alloying. A two-stage mixing technique, i.e., stirring and ultrasonic vibration, was employed to achieve uniform distribution of the reinforcement. Mechanical stirring was performed at 450 rpm for 10 minutes using a stainless-steel impeller, followed by ultrasonic treatment at 20 kHz for 10 minutes, promoting effective deagglomeration and dispersion of the glass particles. The composite slurry was then gravity poured into the preheated cast iron mould, squeezed at a pressure of 130 MPa and allowed to solidify. Fig 1 illustrates the composite fabrication equipment during fabrication.

### 2.3. Characterization techniques

*2.3.1 Mechanical testing*

Vickers microhardness (HV) (following ASTM E384) and Izod impact tests (following ASTM E23) were conducted to evaluate the mechanical behaviour of the fabricated composites. The average of the three readings was considered for each specimen to ensure accuracy and repeatability of the results.

**Fig 2.**
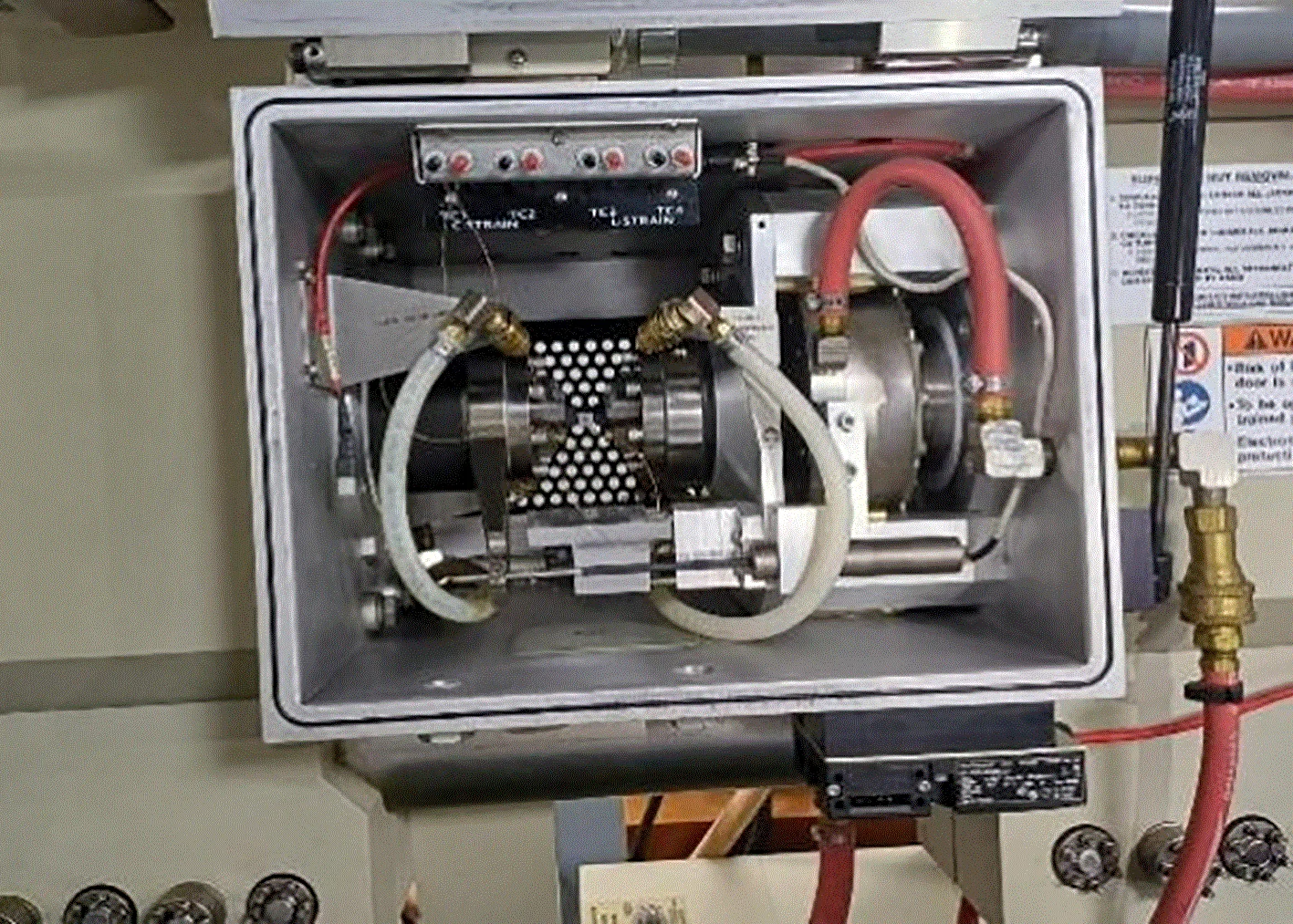
Specimen installation during plain-strain compression test in Gleeble Thermo-Mechanical Simulator.

Cylindrical billet compression tests were conducted using the Gleeble Thermo-Mechanical Simulator (GTMS). The isothermal compressions were conducted at room temperature (30 °C), across a wide range of strain rates: 10 ^−3^, 10 ^−2^, 10 ^−1^, 1, and 10 s ^−1^, to comprehensively evaluate the material’s strain rate sensitivity. Each specimen faced five successive deformation hits at different strain rates, with individual compressive strains of 10%, 10%, 15%, 20%, and 20%, respectively. This strain rate jump (SRJ) approach enabled detailed observation of the material’s flow behaviour and strain rate sensitivity at ambient temperature. The detailed operating parameters considered for the GTMS compression test are listed in Table 2. Fig 2 presents a visual representation of the specimen during the compression test in GTMS.

**Table 2.** Parameters for compression test.

Specimen	Operating Temp. (°C)	Strain rate (s ^−1^) and % of deformation in each HIT				
		HIT-1 (10%)	HIT-2 (10%)	HIT-3 (15%)	HIT-4 (20%)	HIT-5 (20%)
1, 2, 3,4	30	10 ^−3^	10 ^−2^	10 ^−1^	10 ^ °^	10 ^1^

*2.3.2 Tribological testing*

**Fig 3.**
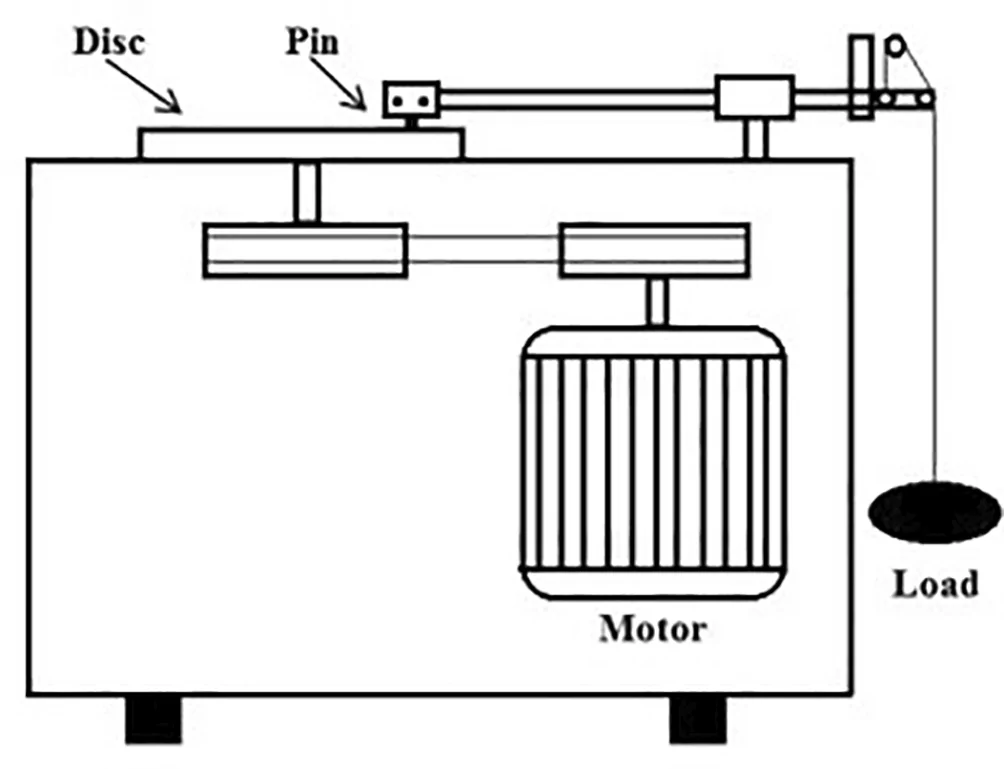
Schematic of the Pin-on-disc test rig.

To evaluate the tribological performance of the fabricated hybrid MMCs, pin-on-disc dry sliding wear tests were performed. Fig 3 illustrates the schematic representation of the pin-on-disc tribometer. To ensure comparability, four composite specimens were tested under identical operating conditions, namely a normal load of 20 N, a sliding velocity of 1 m/s, and a constant sliding distance of 1000 m at room temperature. The counter disc utilized was EN31 grade hardened steel with a hardness of approximately 60 HRC and a surface roughness of 0.1–0.2 μm Ra. The dimensions of the cylindrical pins were maintained at Ø10 × 30 mm. The mass loss was measured before and after the test using a precision balance of 0.1 mg accuracy. The wear rate was estimated in terms of volume loss per sliding distance.

*2.3.3 Microstructural analysis*

Microstructural characterization was performed for the reinforced powders, the fabricated composite specimens, and the worn surfaces to evaluate the morphological features, distribution of reinforcement particles, and wear. The as-received powders were analysed using SEM to assess the particle size and shape. Standard metallographic procedures were followed for sample preparation, including sectioning, grinding, polishing, and etching with Keller’s reagent. The standard compositional proportion of 195 ml H₂O, 5 ml HNO _**3**_, 3 ml HCl, and 2 ml HF are maintained for the preparation of the etchant. The agents of the etchant utilised here were procured from Loba Chemie Pvt. Ltd., Mumbai, India. Elemental analysis was further carried out using Energy Dispersive X-ray Spectroscopy (EDS). The EDS images were attached to the SEM to confirm the distribution and presence of reinforced elements.

## 3. Results and discussion

**Fig 4.**
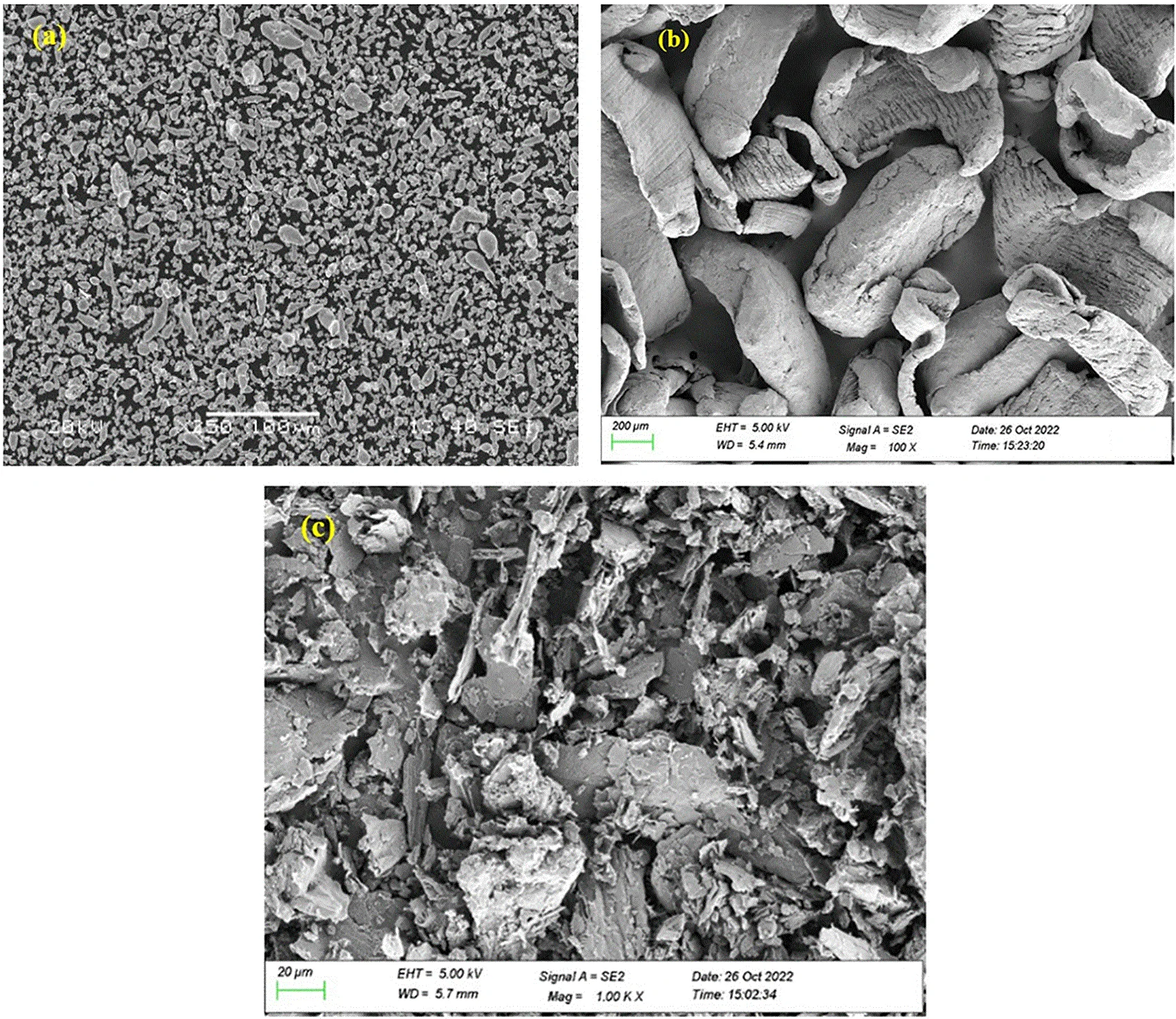
Scanning electron micrographs of powders (a) Mg (b) Zn (c) SLS glass. ^24^

**Fig 5.**
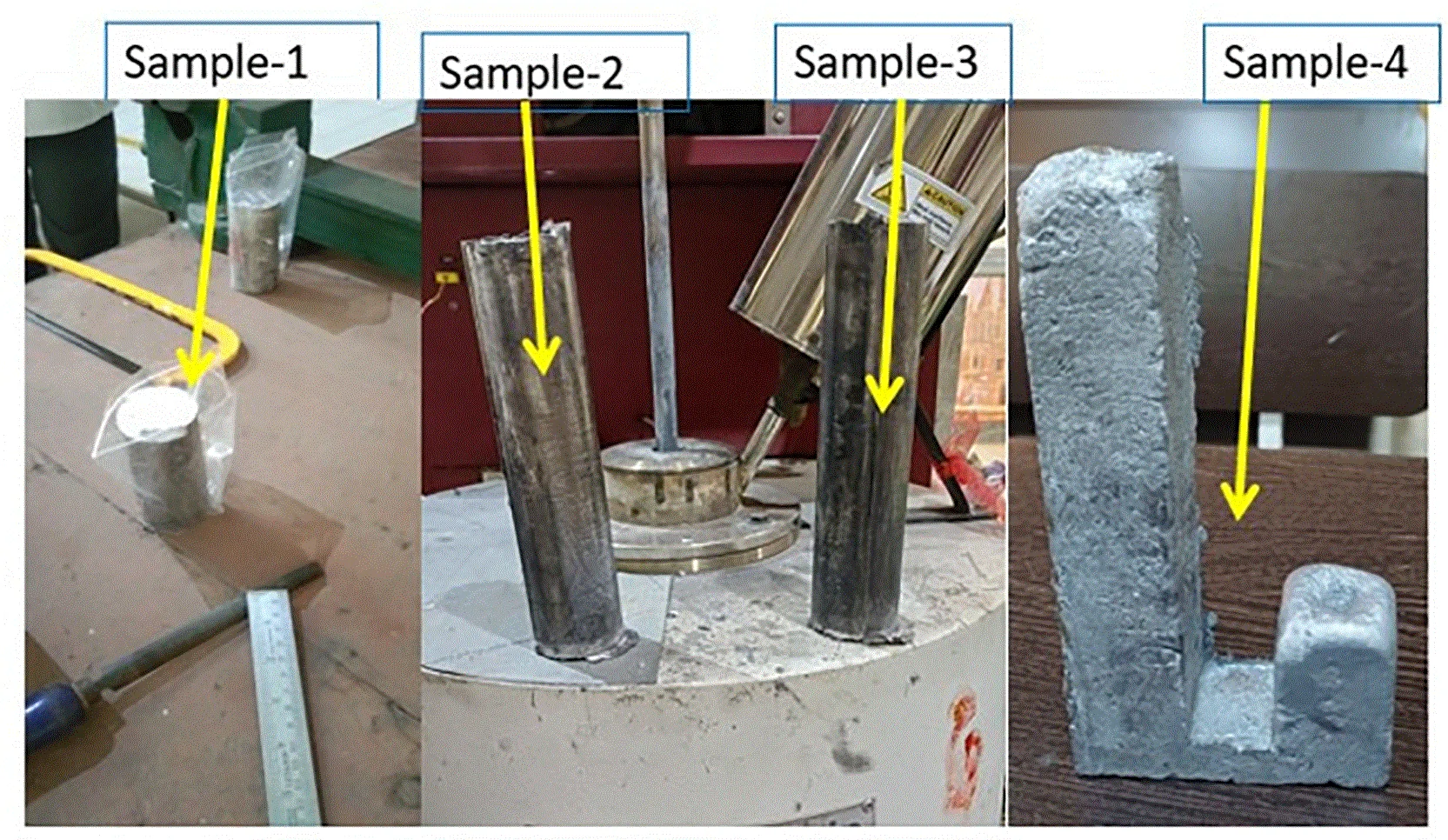
As-cast specimens after fabrication.

The SEM micrographs of the raw powders used for composite fabrication are illustrated in Fig 4 (a) Mg (b) Zn (c) SLS glass. The Mg powder in Fig 4 **(a)** exhibits irregular, flake-like morphology and is larger in size, while the Zn powder in Fig 4 **(b)** appears very fine with rounded to flaky shapes. The soda–lime silica (SLS) glass powder in Fig 4 **(c)** consists of angular particles with a relatively wide range of size distribution. An image analysis software package, ImageJ, is utilised for the analysis of powders. The detailed report is presented in Table 3. Fig 5 illustrates four numbers of fabricated composites obtained after the stir casting process. Specimens were sectioned from the cast ingot for subsequent analysis.

**Table 3.** Physical characteristics of powders.

Powder	Supplier/Prepared	Average size (μm )	Particle shape	Purity/Assay (%)
Aluminium	Loba Chemie	45	Spherical and sub-rounded	98.0
Magnesium		143	Flakey, large in size	99.0
Zinc		20	Rounded and flakey	98.0
Soda-lime silica glass	Mechanical Milling	56	Angular, a wide range of size distribution	

### 3.1. Microstructural observations

**Fig 6.**
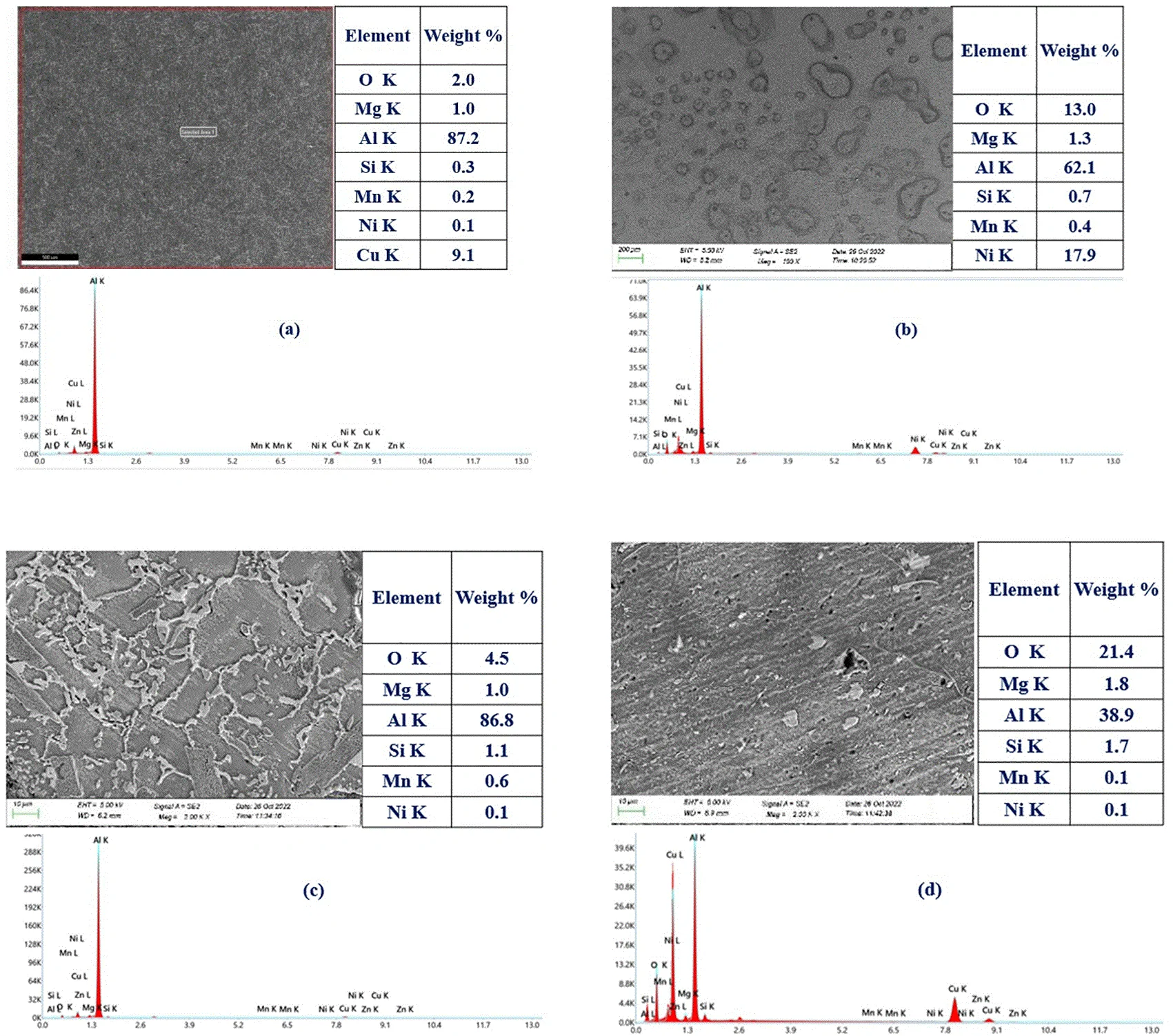
Microstructure of the fabricated MMCs (a) Specimen-1 (b) Specimen-2 (c) Specimen-3 (d) Specimen-4.

A relatively homogeneous distribution of reinforcements within the matrix is observed from the SEM micrographs presented in Fig 6. This indicates efficient dispersion of reinforcements in the matrix achieved through the combined effect of ultrasonic vibration and mechanical stirring. It enhances the load transfer capacity and the overall tribo-mechanical performance of the MMC.

Clustering or segregation of reinforcements were not observed in the micrography. The EDX spectrum confirms the presence of Al, Mg, Zn, Si, O, and Cu, etc. in the fabricated MMC validating the incorporation of alloying and reinforcing elements. The EDX spectra and quantitative data support the microstructural observations, illustrating consistent elemental composition across different regions. The results appear to indicate more homogeneity and effective integration of the reinforcements.

### 3.2. Mechanical properties

*3.2.1 Impact and hardness*

**Fig 7.**
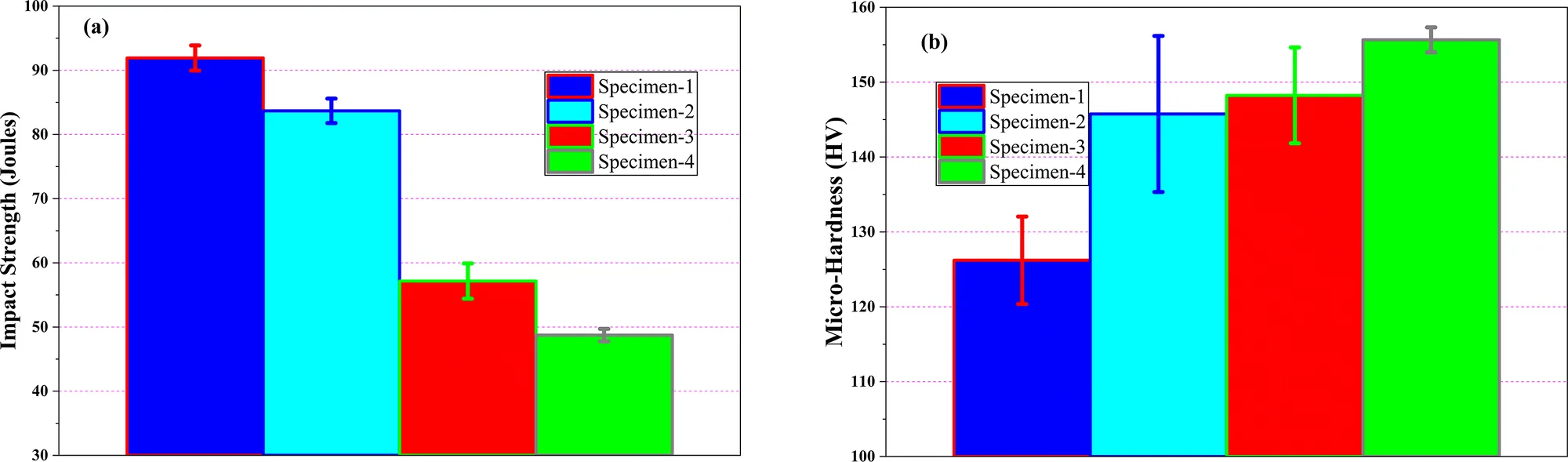
(a) Impact strength and (b) Micro-Hardness.

Impact strength in Joules and Vickers microhardness of the four composites are presented in Fig 7 **(a) and (b)**, respectively. The impact strength of MMCs follows a decreasing trend with increasing reinforcements. Specimen-1 exhibits the highest toughness (~92 J) and specimen 4 the lowest (~48.5 J), reflecting an increasing trend of brittleness with increasing reinforcement. ^14^ In contrast, the microhardness results, presented in Fig 7 **(b)**, increase progressively across the specimens, with Specimen 4 exhibiting the highest hardness value (~155 HV). This trend highlights: higher reinforcement content promotes hardness and brittleness, which reduces impact resistance. ^16^ Specimen with 2% reinforcement offers a balanced combination of impact strength and hardness.

*3.2.2 Compressive strength*

**Fig 8.**
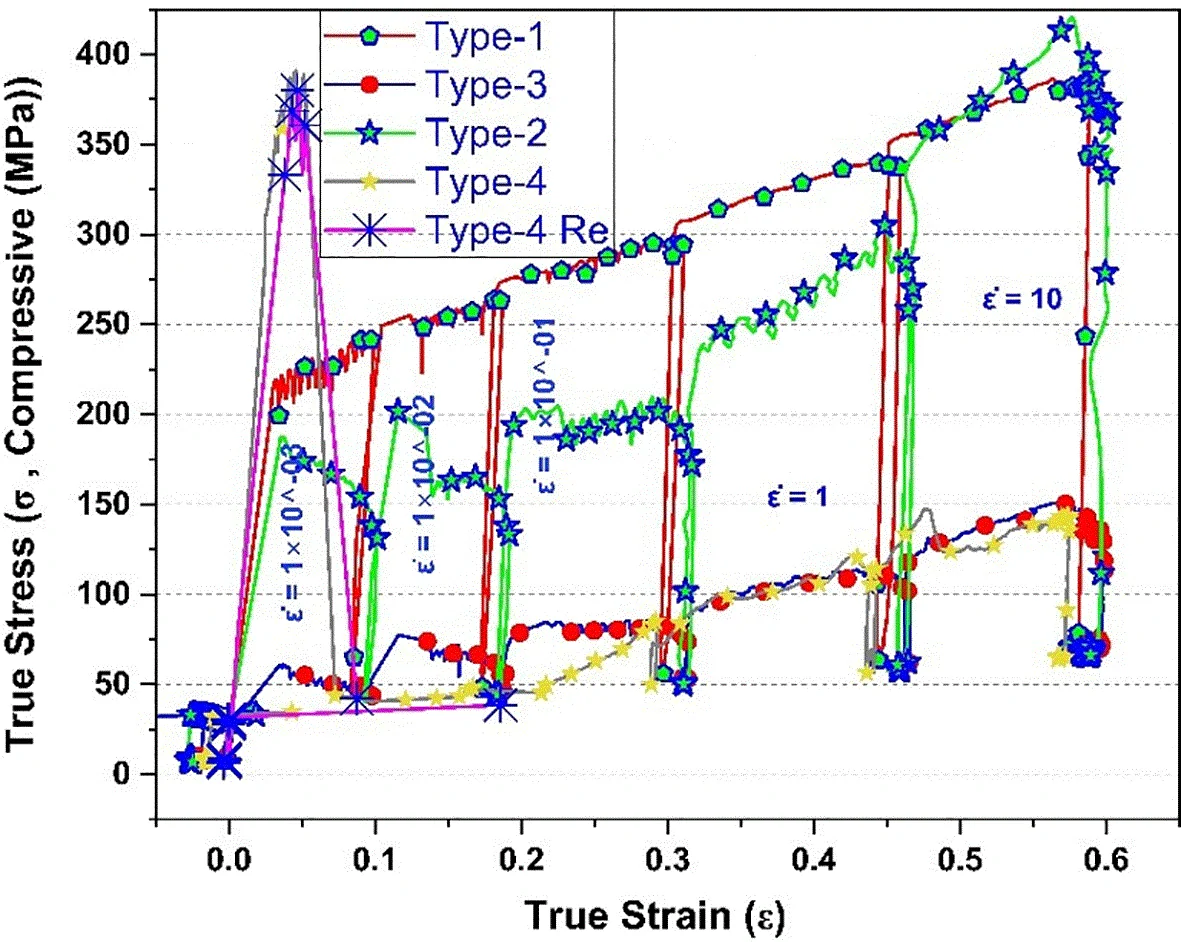
Flow stress plot (True stress vs True strain).

The true stress vs true strain curves obtained from the SRJ compression test in GTMS at 30 °C and varying strain rates (10 ^−3^ to 10 s ^−1^) are presented in Fig 8. At all strain rates, Specimen-1 exhibits the highest compressive strength and strain hardening response across almost all strain rates, elucidating superior resistance to deformation. Specimen-2 also shows good strength and highest strain rate sensitivity. It demonstrates relatively higher flow stress behaviour at higher strain rates ( $\dot{\varepsilon }$ = 1 and 10 s ^−1^). These reveal enhanced thermal softening and homogeneous plastic deformation under rapid loading conditions. Specimen-3 possesses comparably low flow strength and limited energy absorption capacity. Specimen- 4 exhibit high initial flow stress at very low strains, followed by a sudden fall, indicating predominantly a brittle fracture or deformation behaviour. It demonstrates high initial compressive strength, particularly at lower strains and strain rates, suggesting good load-bearing capability. However, its quick fracture highlights brittleness, likely due to particle agglomeration, low interfacial bonding or weak matrix–reinforcement bonding. The test is repeated for Specimen 4 to confirm the result by consistent responses, ensuring the reliability of the observed behaviour.

**Fig 9.**
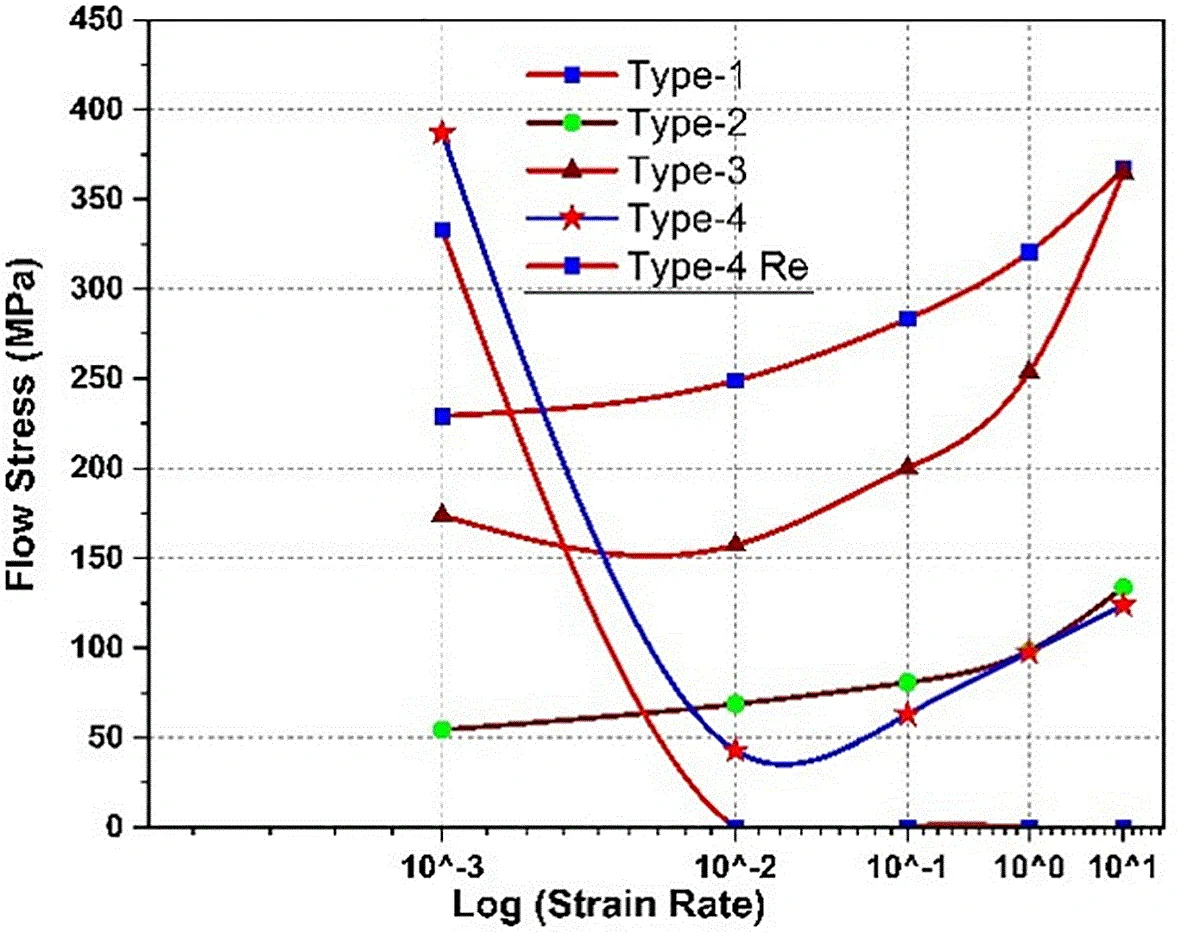
Influence of strain rate on flow stress.

**Fig 10.**
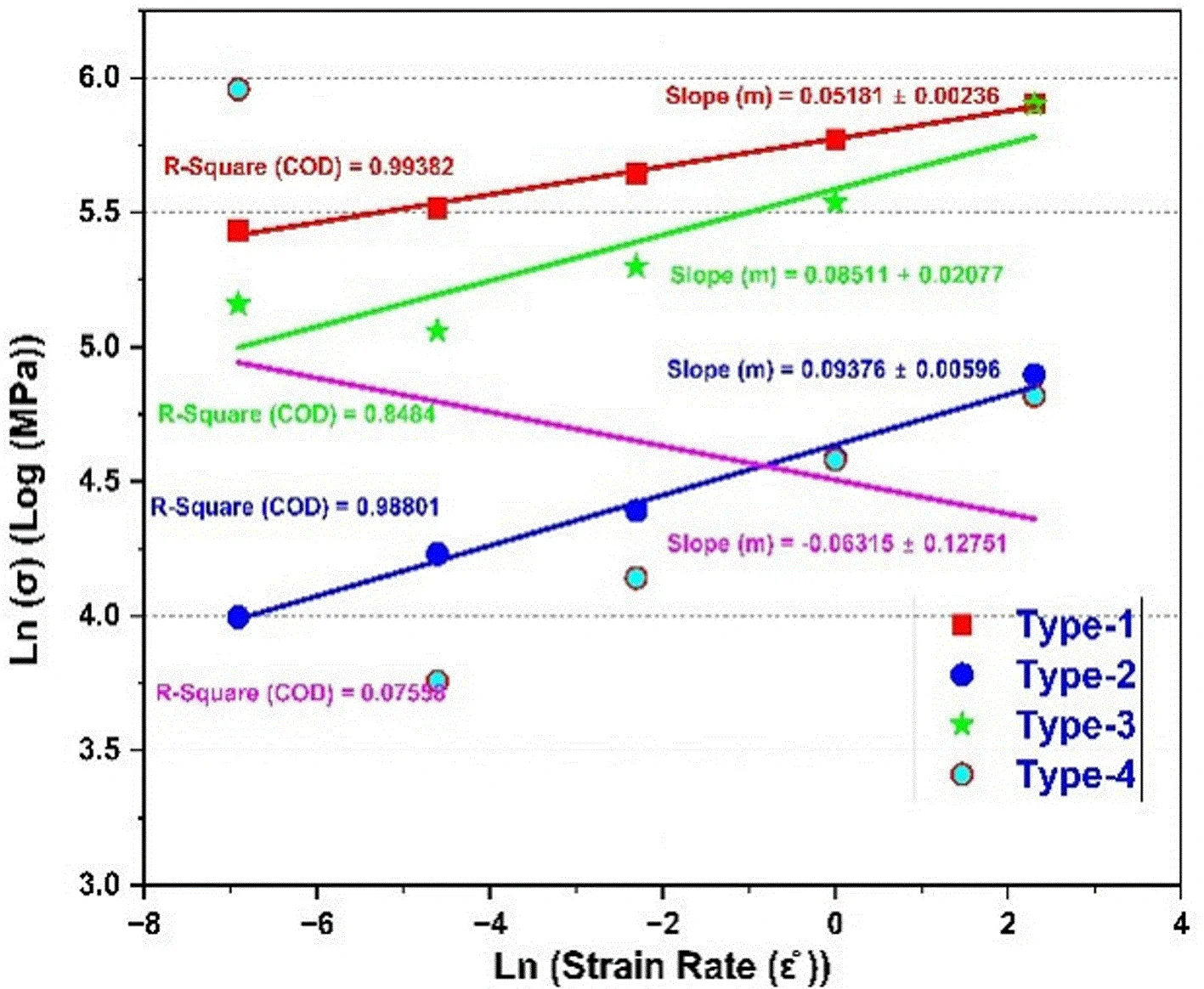
Slope of log-log plot of flow stress vs strain rate.

For finding the relationship between flow stress vs strain rate, the flow stress values (typically at specific strains arbitrarily chosen such as 0.05, 0.15, 0.25, 0.35, 0.5) are extracted and plotted against the corresponding strain rates. Fig 9 illustrates the flow stress vs. strain rate plot for the data for four specimens. The strain rates are shown on a logarithmic scale (Log10), and the corresponding flow stress values are plotted on the Y-axis, indicating the change of flow stress with strain rate. It is observed from the figure that for the operating conditions within the selected range, flow stress rises with strain rate except for specimen type 4. Flow stress progressively decreases for type-3 specimens, indicating thermal softening. For type 4, there is a brittle fracture, hence the plot declines severely. ^24^ ^–^ ^26^ At higher strain rates, dislocations are unable to rearrange or annihilate, which causes a sharp rise in flow stress. Considering the aforesaid strain values, the strain rate sensitivity index (m) was estimated for all kinds of specimens, and the log-log plot between flow stress and strain rate is presented in Fig 10. The strain rate sensitivity is consistently low for all specimens, but its noticeable that with increase in SLS glass percentage up to 4 wt.%, the ‘m’ value increases. The negative ‘m’ value for the type-4 specimen indicates the flow instability and fracture in this case.

### 3.3. Wear properties

**Fig 11.**
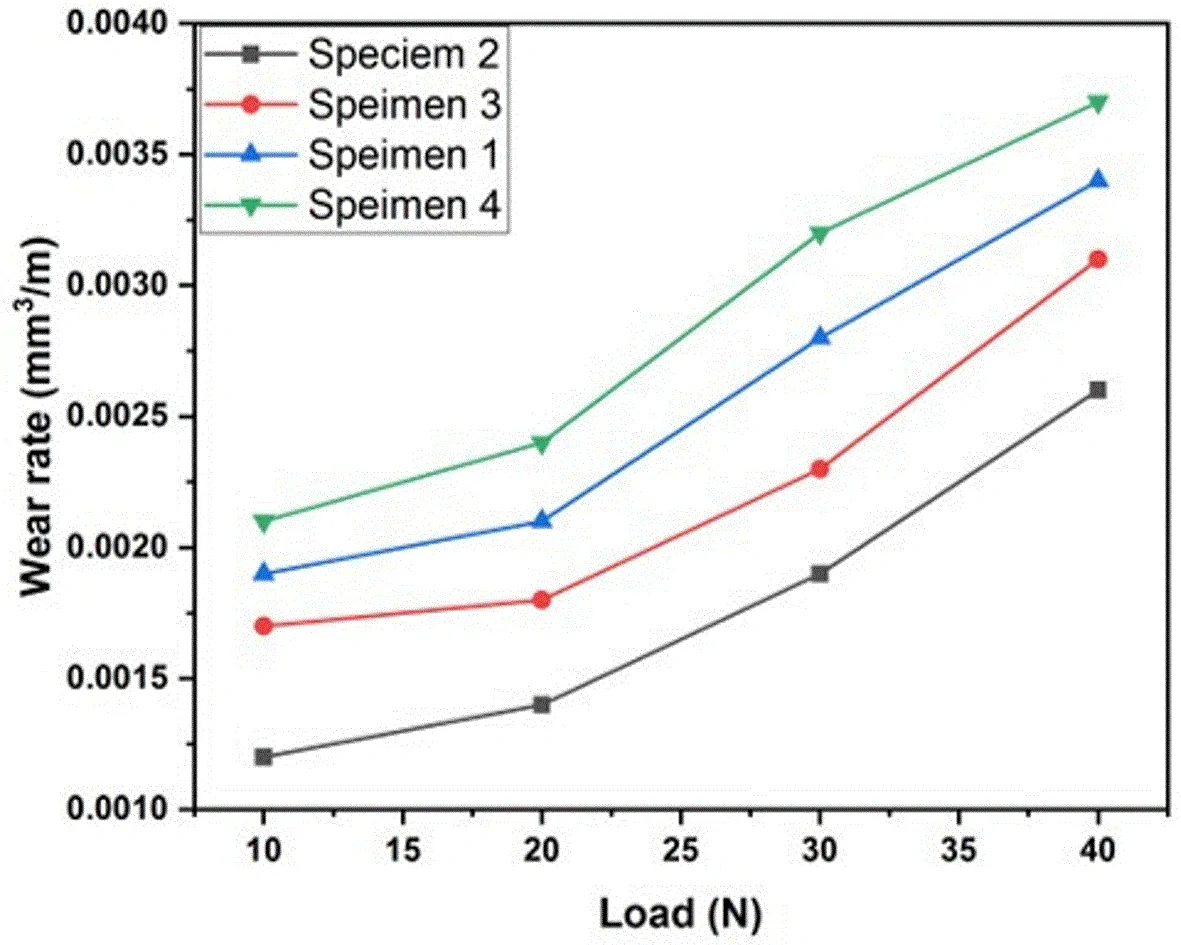
Wear rate of composite samples.

The wear rate of a particular load was determined from the slope of the cumulative volume loss versus sliding distance plot for the composites, obtained using linear least squares fit of the data points. For the wear test, two specimens were tested under identical conditions to find the average wear rate. Figure 11 depicts the wear rate of composites at different applied loads. It can be inferred that specimen 2 exhibited the lowest wear rate. The wear rate increased almost linearly with the load for all the specimens as depicted in Fig 11. It can be explained based on Archard’s law, which states that the wear rate is directly proportional to the applied load, however, it is inversely proportional to the hardness of the softer of the two mating materials. ^27^ It is also evident that specimen 4 shows enhanced wear resistance as compared to other specimens. It can be attributed to the agglomeration of reinforcement particles.

### 3.4. Wear surface morphology

**Fig 12.**
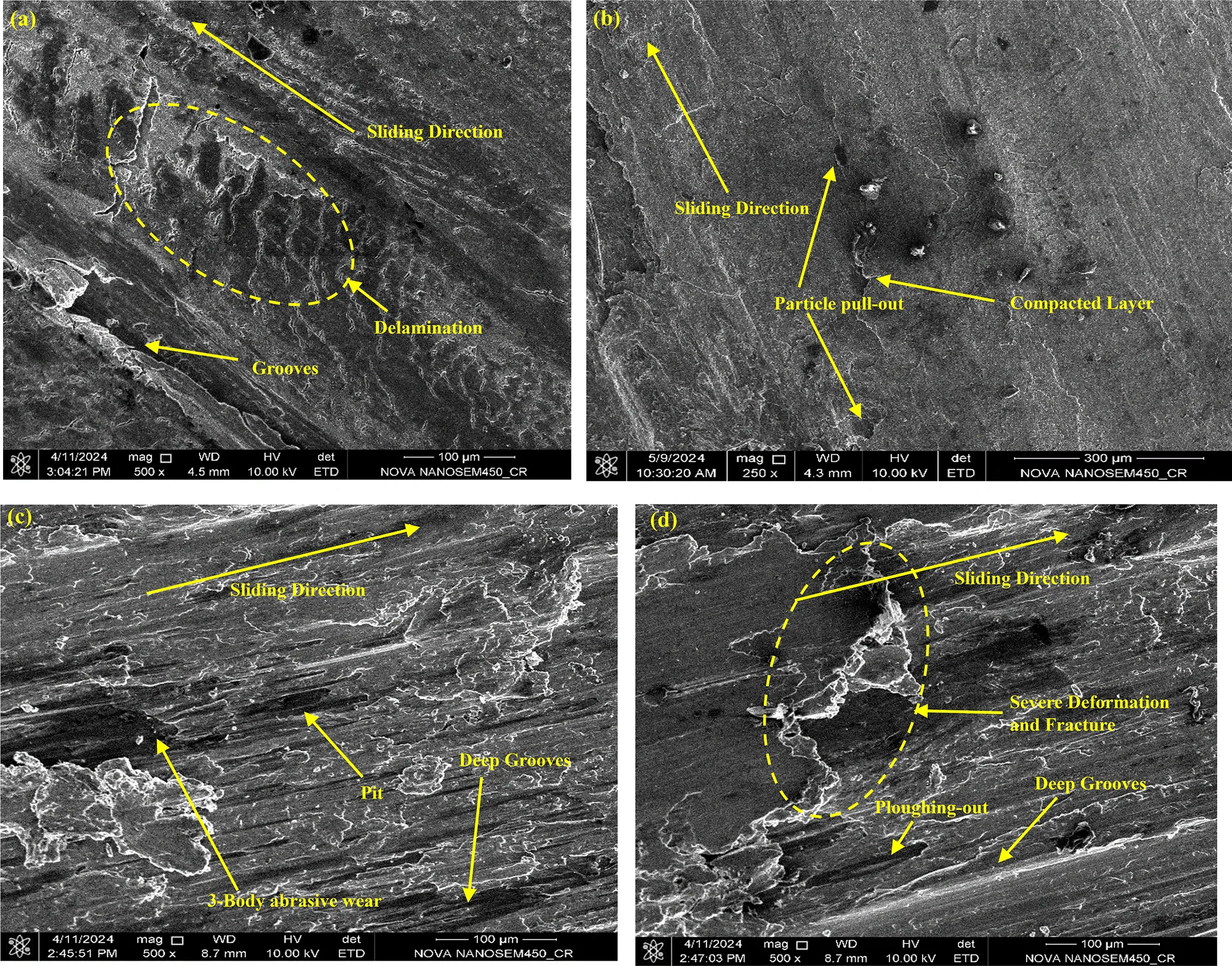
SEM of worn surface morphology (a) Specimen-1 (b) Specimen-2 (c) Specimen-3 (d) Specimen-4.

The SEM images presented in Fig 12 illustrate the worn surface morphologies of the composite specimens after pin-on-disc wear testing. Figure 12 **(a)** shows the sliding direction and smooth, uniform wear tracks with shallow grooves, elucidating mild abrasive wear and minimal material removal in specimen-1. Figure 12 **(b)** presents the worn surface of specimen- 2, where particle pull-out is clearly observed. The detached reinforcement particles contribute to the formation of fine wear debris, which tends to become compacted during sliding and subsequently develops into a mechanically mixed transfer layer at the contact interface. This tribolayer acts as a protective barrier between the sliding surfaces, reducing direct metal-to- metal contact and thereby lowering the material removal. As a result, the wear mechanism shows a transition from dominant abrasive wear towards mild adhesive wear, indicating improved surface stability. The worn surface of specimen- 3, presented in Fig 12 **(c)**, reveals clearer grooves, micro-pits, and particle pull-out regions, substantiating a mix of adhesive and abrasive wear. ^28^ Specimen- 4 in Fig 12 **(d)** illustrates extensive surface damage characterised by severe plastic deformation coupled with prominent deep grooves, propagated cracks, and delamination, which confirms a brittle wear response likely caused by particle agglomeration and weak interfacial bonding [29,30]. The higher reinforcement contents beyond an optimum level led to deteriorated wear performance due to reduced ductility and poor structural integrity.

## 4. Conclusions

Based on the extensive analysis on the fabrication and tribo-mechanical characterization of soda-lime silica glass reinforced Al-Mg-Zn Matrix composite, the following conclusions can be drawn:

• The hybrid aluminium-based composites reinforced with soda-lime silica glass and alloyed with Mg and Zn were successfully fabricated using vibration-supported stir-squeeze casting technique, with varying reinforcement levels (1, 2, 4, and 5 wt.%).
• Among all compositions, the composite with 2 wt.% reinforcement exhibited the balance of mechanical and tribological properties, including good impact strength, moderate hardness, and strain rate sensitive flow behaviour under compression.
• Higher reinforcement content increased hardness, with Specimen 4 showing the maximum, a marked reduction in impact strength due to brittleness.
• Gleeble compression tests revealed that specimen 3 and 4 exhibited high initial flow stress but failed prematurely, indicating brittle deformation arising from inadequate interfacial bonding and agglomeration.
• A transition from predominantly mild adhesive wear in specimen 1 to abrasive, delamination, and micro-fracture–assisted wear in specimen 4 is observed, indicating that higher reinforcement content enhances surface damage and deteriorates wear performance.

## Data Availability

The datasets generated and/or analysed during the current study are publicly available in the Figshare repository under the Creative Commons Attribution 4.0 International (CC BY 4.0) license and are accessible at: DOI: 10.6084/m9.figshare.32336223. ^29^ **Repository name:** *Enhancement of Tribological and Mechanical Properties via Soda-Lime Silica Glass Reinforcement in Al-Mg-Zn Matrix Composite.* https://doi.org/10.6084/m9.figshare.32336223. ^29^ The project contains the following underlying data: Underlying- Fig 1.jpg (the figure presented in Fig 1). Underlying- Fig 2.jpg (the figure presented in Fig 2). Underlying- Fig 3.jpg (the figure presented in Fig 3). Underlying- Fig 4. (a).jpg (the figure presented in Fig 4. (a)). Underlying- Fig 4. (b).jpg (the figure presented in Fig 4. (b)). Underlying- Fig 4. (c).tif (the figure presented in Fig 4. (c)). Underlying- Fig 5.jpg (the figure presented in Fig 5). Underlying- Fig 6. (a).jpg (the figure presented in Fig 6. (a)). Underlying- Fig 6. (b).jpg (the figure presented in Fig 6. (b)). Underlying- Fig 6. (c).jpg (the figure presented in Fig 6. (c)). Underlying- Fig 6. (d).jpg (the figure presented in Fig 6. (d)). Underlying- Fig 7. (a).jpg (the figure presented in Fig 7. (a)). Underlying- Fig 7. (b).jpg (the figure presented in Fig 7. (b)). Underlying- Fig 8.jpg (the figure presented in Fig 8). Underlying- Fig 9.jpg (the figure presented in Fig 9). Underlying- Fig 10.jpg (the figure presented in Fig 10). Underlying- Fig 11.jpg (the figure presented in Fig 11). Underlying- Fig 12. (a).jpg (the figure presented in Fig 12. (a)). Underlying- Fig 12. (b).jpg (the figure presented in Fig 12. (b)). Underlying- Fig 12. (c).jpg (the figure presented in Fig 12. (c)). Underlying- Fig 12. (d).jpg (the figure presented in Fig 12. (d)). Underlying_RAW_impact strength and hardnes.xlxs (the raw data containing hardness and impact strength of all tests and standard deviations). Extended_Raw _gleeble compression test.xlsx (this file contains the raw information from gleeble compression tests and the calculations of all the plots. This may be considered for other analysis/extended analysis). No extended data is associated with this article.
