# Supplementary material for: Enhancement of Tribological and Mechanical Properties via Soda-Lime Silica Glass Reinforced Al-Mg-Zn Matrix Composite
Source: F1000Res. 2026 Aug 20;15:1181. Originally published 2026 Jul 17. [Version 2] doi: 10.12688/f1000research.183177.2 (PMC13560067; doi:10.12688/f1000research.183177.2)
Supplement: Peer review history

# Reviewer report: Reviewer response for version 2

**DOI:** 10.5256/f1000research.208263.r513111

**Published:** 2026-08-28

**Authors:** Akhtar khan

**Affiliations:** 1 Indian Institute of Information Technology Design and Manufacturing Kurnool, Kurnool, Andhra Pradesh, India

The manuscript is revised and can be accepted in its present form.

Is the work clearly and accurately presented and does it cite the current literature?

Yes

If applicable, is the statistical analysis and its interpretation appropriate?

Not applicable

Are all the source data underlying the results available to ensure full reproducibility?

No source data required

Is the study design appropriate and is the work technically sound?

Yes

Are the conclusions drawn adequately supported by the results?

Yes

Are sufficient details of methods and analysis provided to allow replication by others?

Yes

Reviewer Expertise:

Manufacturing Science and Technology

I confirm that I have read this submission and believe that I have an appropriate level of expertise to confirm that it is of an acceptable scientific standard.

# Reviewer report: Reviewer response for version 1

**DOI:** 10.5256/f1000research.202191.r503396

**Published:** 2026-08-06

**Authors:** Rashmi Ranjan Das

**Affiliations:** 1 Indian Institute of Technology Dhanbad, Dhanbad, Jharkhand, India

**Enhancement of Tribological and Mechanical Properties via Soda-Lime Silica Glass Reinforced Al-Mg-Zn Matrix Composite**

**General comments**

 The present research addresses a relevant topic in the field of aluminum-based hybrid metal matrix composites and presents a systematic experimental investigation on the influence of soda-lime silica (SLS) glass reinforcement on the microstructural, mechanical, and tribological properties of Al–Mg–Zn composites. The manuscript is generally well organized, and the experimental methodology is described with sufficient clarity. The results are presented in a logical sequence and are supported by appropriate characterization techniques. However, the manuscript would benefit from minor revisions to strengthen scientific discussion, improve the articulation of the novelty and research gap, and enhance the clarity and readability of certain sections. *These revisions are primarily editorial in nature and are expected to improve the overall quality and impact of the manuscript without requiring additional experimental investigations **.***

**Introduction Section**

- The research gap should be more explicitly articulated, highlighting the unresolved issues in SLS glass-reinforced Al–Mg–Zn composites and how the present work addresses them.
- Novelty of the study should be stated more clearly at the end of the introduction, emphasizing the contribution of both the material system and the vibration-supported stir-squeeze casting process.
- A brief discussion on advantages of the selected processing route over conventional fabrication techniques would help justify its adoption.
- The introduction would benefit from minor language editing and a more critical synthesis of the literature, rather than a sequential description of previous studies.

**Materials and methods**

- The authors may briefly justify the selection of the key processing parameters (e.g., stirring speed, ultrasonic treatment duration, and squeeze pressure) by citing relevant literature or stating that these were adopted from established processing practices.
- Please specify the particle size (or particle size range) of the Mg, Zn, and SLS glass powders to further enhance the reproducibility of the fabrication procedure.
- A minor language revision is recommended to improve readability and grammatical consistency throughout this section.

**Characterization techniques**

 The characterization methodology is comprehensive and well organized. The testing conditions for the mechanical, tribological, and microstructural analyses are described with sufficient detail to facilitate reproducibility. However, the below mentioned points can be taken care of:

- Please specify the applicable testing standards (e.g., ASTM/ISO) for the hardness, impact, compression, and wear tests, wherever relevant, to improve the completeness of the experimental methodology.
- The authors may briefly mention the number of specimens tested for the compression and wear experiments (if different from the hardness tests) to clarify the repeatability of the reported results.
- A minor language revision is recommended to improve readability and maintain consistency in the presentation of units, symbols, and terminology throughout this section.

**Results and discussion**

 The Results and Discussion section is logically organized, and the experimental observations are generally correlated with the microstructural features and mechanical/tribological behavior. The discussion provides a clear description of the observed trends and is supported by relevant figures. However there are a few concerns noted below:

- The discussion would benefit from a clearer explanation of the underlying mechanisms responsible for the observed trends (e.g., hardness, impact strength, compressive behavior, and wear resistance), rather than primarily describing experimental observations.
- Wherever trends are discussed, please refer more explicitly to the corresponding figures (e.g., Fig. 7–12) to improve the continuity and readability of the discussion.
- Some statements, such as "homogeneous distribution", "no clustering observed", and "effective integration of reinforcements", appear qualitative. The authors may consider using more cautious wording (e.g., "appears relatively homogeneous within the observed regions") unless these observations are representative of the entire specimen.
- The Results and Discussion section would benefit from minor language editing to improve clarity, remove repetitive statements, and enhance the overall flow of the discussion.

Is the work clearly and accurately presented and does it cite the current literature?

Yes

If applicable, is the statistical analysis and its interpretation appropriate?

Yes

Are all the source data underlying the results available to ensure full reproducibility?

Yes

Is the study design appropriate and is the work technically sound?

Yes

Are the conclusions drawn adequately supported by the results?

Yes

Are sufficient details of methods and analysis provided to allow replication by others?

Partly

Reviewer Expertise:

1. Fracture Mechanics: Computational And Experimental, 2. Material Fracture Analyses: Isotropic (Metals), Orthotropic (FRP Composites), Anisotropic (FGMs & Rocks); 3. Numerical Methods: Finite Element Method, 4. Structural Design: Adhesive Bonded Joints, FRP/Epoxy Patch/coating, HEMM Components, PVC/FRP Pipe Joints, Masonry Structures

I confirm that I have read this submission and believe that I have an appropriate level of expertise to confirm that it is of an acceptable scientific standard.

# Response

**Published:** 2026-08-07

**Authors:** Dr. Pushkar Jha

**Affiliations:** School of Mechanical Engineeri, Kalinga Institute of Industrial Technology School of Mechanical Engineering, Bhubaneswar, Odisha, India

The authors sincerely thank the reviewers for their valuable comments and constructive suggestions. All comments have been carefully considered, and the manuscript has been revised accordingly. The point-by-point responses are provided below.

**Introduction Section**

**Response 1: **Thank you for the valuable suggestion. The research gap has been more explicitly articulated in the revised manuscript, highlighting the unresolved issues in SLS glass-reinforced Al–Mg–Zn composites and how the present study addresses them.

**Response 2: **Thank you for the insightful comment. The novelty of the present study has been clearly stated at the end of the Introduction, emphasizing both the developed material system and the vibration-supported stir-squeeze casting process.

**Response 3: **Thank you for the valuable suggestion. The discussion indicates the advantages of the selected processing route over conventional fabrication techniques.

**Response 4: **Thank you for the valuable suggestion. The Introduction has been carefully reviewed and revised to improve language, readability, and the overall presentation of the literature. The corresponding changes have been highlighted in the revised manuscript.

**Materials and Methods**

**Response 5: **Thank you for the valuable suggestion. The selected processing parameters are commonly adopted in the literature.

**Response 6: **Thank you for the valuable comment. The particle size and shape of the Mg, Zn, and SLS glass powders are already presented in **Table 3**.

**Response 7: **Thank you for the valuable suggestion. The Materials and Methods section has been carefully reread and revised to improve readability and grammatical consistency. The revised text has been highlighted in the manuscript for the reviewer's reference.

**Characterization Techniques**

**Response 8: **Thank you for the valuable suggestion. The applicable testing standards (ASTM/ISO), wherever relevant, have been included in the revised manuscript.

**Response 9: **Thank you for the valuable comment. The number of specimens tested for the wear experiments has been specified in the revised manuscript. For the compression test, only **Specimen 4** was retested to verify the observed result, and this information was already provided in the manuscript.

**Response 10: **Thank you for the valuable suggestion. The section has been carefully reviewed and revised to improve readability and to maintain consistency in the presentation of units, symbols, and terminology. The corresponding changes have been highlighted in the revised manuscript for the reviewer's reference.

**Results and Discussion**

**Response 11: **Thank you for the valuable suggestion. The discussion has been strengthened through careful revision and the inclusion of additional supporting explanations.

**Response 12: **Thank you for the valuable suggestion. The discussion has been presented in a composition-wise manner, with the relevant figures cited appropriately.

**Response 13: **Thank you for the valuable suggestion. The statements have been modified with more appropriate and cautious wording in **Section 3.1** of the revised manuscript.

**Response 14: **Thank you for the valuable suggestion. The Results and Discussion section has been thoroughly reviewed and revised to improve clarity, eliminate repetitive statements, and enhance the overall flow of the discussion. The corresponding changes have been highlighted in the revised manuscript.

# Reviewer report: Reviewer response for version 1

**DOI:** 10.5256/f1000research.202191.r503399

**Published:** 2026-08-03

**Authors:** Santosh Kumar Sahu

**Affiliations:** 1 VIT-AP University, Amaravati, Andhra Pradesh, India

The manuscript presents an interesting investigation on the developed composite material and its corresponding mechanical and tribological behaviour. The experimental methodology and characterization approach adopted by the authors are appreciable, and the study contributes useful information to the field of metal matrix composites. The manuscript may be considered for indexing after minor revision.

 The following suggestions may further improve the quality and clarity of the manuscript:

 1.    The use of argon gas shielding during processing is appreciable and necessary to minimize oxidation. However, the flow rate/supply rate of argon gas has not been mentioned. This information should be included so that future researchers may use it as a processing reference. 

 2.    The figure captions should be more descriptive and informative. Presently, captions of Fig. 2 and Fig. 3 appear incomplete and insufficiently explained. Proper figure descriptions are recommended for better understanding of the presented results. 

 3.    The operating/test temperature during the wear experiments has not been specified. Since wear behaviour is temperature dependent, the corresponding testing temperature should be clearly mentioned in the manuscript. 

 4.    In Section 3.2.1, it is stated that Specimen-1 exhibits the highest toughness whereas Specimen-4 shows the lowest toughness. However, the corresponding quantitative values are not clearly reported. The authors are requested to provide the exact toughness values for proper comparison.

Is the work clearly and accurately presented and does it cite the current literature?

Yes

If applicable, is the statistical analysis and its interpretation appropriate?

Yes

Are all the source data underlying the results available to ensure full reproducibility?

Yes

Is the study design appropriate and is the work technically sound?

Yes

Are the conclusions drawn adequately supported by the results?

Yes

Are sufficient details of methods and analysis provided to allow replication by others?

Yes

Reviewer Expertise:

Materials

I confirm that I have read this submission and believe that I have an appropriate level of expertise to confirm that it is of an acceptable scientific standard.

# Response

**Published:** 2026-08-07

**Authors:** Dr. Pushkar Jha

**Affiliations:** School of Mechanical Engineeri, Kalinga Institute of Industrial Technology School of Mechanical Engineering, Bhubaneswar, Odisha, India

The authors sincerely thank the reviewers for their valuable comments and constructive suggestions. All comments have been carefully considered, and the manuscript has been revised accordingly. The point-by-point responses are provided below.

**Response 1: **Thank you for the valuable suggestion. The mixture gas flow rate used during the processing has been included in the revised manuscript.

**Response 2: **Thank you for the valuable comment. The captions of Figures 2 and 3 have been revised to provide more descriptive and informative explanations in the revised manuscript.

**Response 3: **Thank you for the valuable suggestion. The operating temperature during the wear experiments has been included in the revised manuscript.

**Response 4: **Thank you for the valuable comment. The quantitative toughness values corresponding to Specimen-1 and Specimen-4 have been included in the revised manuscript for better comparison.

# Reviewer report: Reviewer response for version 1

**DOI:** 10.5256/f1000research.202191.r503391

**Published:** 2026-07-28

**Authors:** Akhtar khan

**Affiliations:** 1 Indian Institute of Information Technology Design and Manufacturing Kurnool, Kurnool, Andhra Pradesh, India

The manuscript entitled “Enhancement of Tribological and Mechanical Properties via Soda-Lime Silica Glass Reinforced Al-Mg-Zn Matrix Composite” is recommended for indexing after minor revision, considering the relevance of the work in the field of metal matrix composites and tribological performance enhancement. The study presents useful findings regarding the influence of Soda-Lime Silica (SLS) glass reinforcement on the mechanical and wear behaviour of the developed composite material.

 The following suggestions may further improve the manuscript:

 1.    The preparation method and particle size details of the SLS glass reinforcement used for casting are missing. Since these parameters significantly influence composite properties, the authors are requested to include the preparation procedure and size specifications of the reinforcement particles. 

 2.    In the stir casting process, the stirring frequency/speed and the duration of stirring have not been mentioned. These processing parameters are essential for reproducibility and uniform reinforcement distribution; therefore, the corresponding details should be included in the manuscript.

 3.    In Section 3.2.2, the justification behind the failure of the fourth specimen should be discussed in detail. Including the possible causes and failure mechanism would further strengthen the technical quality of the manuscript.

Is the work clearly and accurately presented and does it cite the current literature?

Yes

If applicable, is the statistical analysis and its interpretation appropriate?

Not applicable

Are all the source data underlying the results available to ensure full reproducibility?

No source data required

Is the study design appropriate and is the work technically sound?

Yes

Are the conclusions drawn adequately supported by the results?

Yes

Are sufficient details of methods and analysis provided to allow replication by others?

Yes

Reviewer Expertise:

Manufacturing Science and Technology

I confirm that I have read this submission and believe that I have an appropriate level of expertise to confirm that it is of an acceptable scientific standard, however I have significant reservations, as outlined above.

# Response

**Published:** 2026-08-07

**Authors:** Dr. Pushkar Jha

**Affiliations:** School of Mechanical Engineeri, Kalinga Institute of Industrial Technology School of Mechanical Engineering, Bhubaneswar, Odisha, India

The authors sincerely thank the reviewers for their valuable comments and constructive suggestions. All comments have been carefully considered, and the manuscript has been revised accordingly. The point-by-point responses are provided below.

**Response1: **Thank you for the valuable suggestion. The preparation method and particle size details of the SLS glass reinforcement have been included in the revised manuscript.

**Response 2: **Thank you for the valuable comment. The stirring speed and stirring duration employed during the stir casting process have been included in the revised manuscript.

**Response 3: **Thank you for the insightful suggestion. The possible reasons and failure mechanism of the fourth specimen have been discussed in the revised manuscript.
